# The Developmental Trajectory of Empathy and Its Association with Early Symptoms of Psychopathology in Children with and without Hearing Loss

**DOI:** 10.1007/s10802-021-00816-x

**Published:** 2021-04-07

**Authors:** Yung-Ting Tsou, Boya Li, Carin H Wiefferink, Johan H M Frijns, Carolien Rieffe

**Affiliations:** 1 Unit of Developmental and Educational Psychology, Institute of Psychology, Leiden University, Leiden, The Netherlands; 2 Dutch Foundation for the Deaf and Hard of Hearing Child, Amsterdam, The Netherlands; 3 Department of Otorhinolaryngology and Head & Neck Surgery, Leiden University Medical Center, Leiden, The Netherlands; 4 Leiden Institute for Brain and Cognition, Leiden University, Leiden, The Netherlands; 5 Department of Human Media Interaction, Faculty of Electrical Engineering, Mathematics and Computer Science, University of Twente, Enschede, The Netherlands; 6 Department of Psychology and Human Development, Institute of Education, University College London, London, UK

**Keywords:** Hearing loss, sensorineural, Empathy, Psychopathology, Child development, Longitudinal study, Cochlear implant

## Abstract

Empathy enables people to share, understand, and show concern for others’ emotions. However, this capacity may be more difficult to acquire for children with hearing loss, due to limited social access, and the effect of hearing on empathic maturation has been unexplored. This four-wave longitudinal study investigated the development of empathy in children with and without hearing loss, and how this development is associated with early symptoms of psychopathology. Seventy-one children with hearing loss and cochlear implants (CI), and 272 typically-hearing (TH) children, participated (aged 1–5 years at Time 1). Parents rated their children’s empathic skills (affective empathy, attention to others’ emotions, prosocial actions, and emotion acknowledgment) and psychopathological symptoms (internalizing and externalizing behaviors). Children with CI and TH children were rated similarly on most of the empathic skills. Yet, fewer prosocial actions were reported in children with CI than in TH children. In both groups, affective empathy decreased with age, while prosocial actions and emotion acknowledgment increased with age and stabilized when children entered primary schools. Attention to emotions increased with age in children with CI, yet remained stable in TH children. Moreover, higher levels of affective empathy, lower levels of emotion acknowledgment, and a larger increase in attention to emotions over time were associated with more psychopathological symptoms in both groups. These findings highlight the importance of social access from which children with CI can learn to process others’ emotions more adaptively. Notably, interventions for psychopathology that tackle empathic responses may be beneficial for both groups, alike.

**Supplementary Information:**

The online version contains supplementary material available at 10.1007/s10802-021-00816-x.

Empathy is the capacity to share and understand other people’s emotions, and to affectively and appropriately respond to those emotions (Hoffman, 1990; Rieffe et al., 2010). This capacity is essential for successfully navigating daily social life, given its role as the “social glue” in stimulating social belongingness (De Waal, 2009). Higher levels of empathy are associated with better social competence and fewer symptoms of internalizing and externalizing problems (e.g., Mayberry & Espelage, 2007; Pursell et al., 2008; Smith, 2015; Tully & Donohue, 2017). Yet despite its importance in children’s psychosocial wellbeing, very little is known about the development of such capacity in children with a cochlear implant (CI), who are at risk for experiencing difficulties in social participation during early childhood as a result of hearing loss (Bat-Chava & Deignan, 2001; Calderon & Greenberg, 2012; Punch & Hyde, 2011; Rieffe et al., 2015). The current four-wave study attempted to discover how empathy develops in children with a CI, and how this development is associated with early symptoms of psychopathology across the preschool years, by using a longitudinal design for the first time.

For the maturation of empathy, four skills are involved during the preschool years. According to Hoffman (1990), empathy starts with an affective mirroring of other people’s emotions during the first days of life. This affective component of empathy triggers emotional arousal in the person witnessing an emotional display, allowing that individual to feel what others are feeling (Hatfield et al., 1993; Hoffman, 1990). A newborn tends to experience an overwhelming level of personal emotional arousal when witnessing someone in distress because they are not yet able to differentiate between themselves and another person. However from the age of one year on, children become more aware of other people’s emotional displays, and experience a lower level of personal arousal (Hoffman, 1990; Rieffe et al., 2010). This enables a child to shift their attention away from their own arousal to the person who is experiencing the emotion. Paying attention to other people is the starting point for understanding how others feel. As a child’s responses to others’ emotions increase, they may start to show concern for other people through prosocial actions, for example by comforting, helping, or sharing (Hoffman, 1990; Rieffe et al., 2010). An early form of such prosocial actions can be observed even in two-year-old children (Zahn-Waxler et al., 1992). Moreover, being able to acknowledge other people’s emotions is a prerequisite for understanding the causes of those feelings. This skill starts developing as early as four months old, but it is not mastered until middle childhood (Durand et al., 2007; Montague & Walker-Andrews, 2001).

For young children with hearing loss living in a predominantly hearing social environment, the acquisition of these empathic skills is not easy. Children need social exposure and participation to master these skills for attending to and understanding others’ emotions, and for reacting appropriately to them (Rieffe et al., 2015). However, children with hearing loss in a predominantly hearing world are given fewer chances to observe or participate in social interactions, due to communicative difficulties. They also miss out on a variety of information relevant for learning the meaning of emotions, such as the sound of other crying babies, emotion expressions displayed behind them, and conversations not directed to them. Such information represents sources of incidental learning, or unplanned, unintended, and unprompted learning (Kelly, 2012). Incidental learning is important for the acquisition of social-emotional skills (Moeller, 2007).

Even within their family, children with hearing loss face challenges in dyadic interactions from birth because over 90% of them are born to hearing families (Mitchell & Karchmer, 2004), and parents with typical hearing often know less well how to attract attention or communicate with a child with hearing loss (Calderon & Greenberg, 2012). This often results in a more directive and protective parenting style, with less turn-taking and shorter utterances in conversations, and less usage of mental-state language (Dirks et al., 2020; Morgan et al., 2014; Pinquart, 2013). Although CIs significantly improve many deaf children’s hearing and speech performance (Waltzman, 2006), these children still experience difficulties following conversations when there are background noises or more than one talker, due to the congenital hearing loss and the technological limitations of the hearing devices (e.g., Caldwell & Nittrouer, 2013; Cullington & Zeng, 2008; Misurelli & Litovsky, 2015). Moreover, parents and other family members can easily overestimate the hearing ability of a child with a CI.

To date, our knowledge regarding empathic maturation in the population with hearing loss is scarce. In a study that measured overall empathy levels using teacher reports, children with hearing loss aged 4 to 12 years were rated lower than their typically hearing (TH) peers (Peterson, 2016). When different types of empathic skills were investigated separately, the results were mixed. The levels of affective empathy were not found to be different between children with and without hearing loss (Dirks et al., 2017; Netten et al., 2015). Yet, parent reports and self-reports indicated that children with hearing loss showed fewer prosocial actions (Dirks et al., 2017; Netten et al., 2015) while looking more often at the person experiencing an emotion than TH children during an observational task (Netten et al., 2015). Preschoolers with a CI also exhibited difficulties in acknowledging others’ emotion expressions (Wang et al., 2019; Wang et al., 2016).

A longitudinal account of empathy is highly relevant to our knowledge of social-emotional development, as empathy is consistently found to play a protective role in typical development. For example, a higher level of empathy is associated with fewer internalizing symptoms, such as depression and anxiety (e.g., Smith, 2015; Tully & Donohue, 2017), and with fewer externalizing behaviors, such as aggression and conduct problems (e.g., Mayberry & Espelage, 2007; Pursell et al., 2008). According to two longitudinal studies in typically developing preschool children, this negative association between empathy (measured as an overall response) and behavioral difficulties is stable from preschool to early primary school years (Hastings et al., 2000; Zhou et al., 2002), suggesting that empathy is effective in reducing behavioral problems. When children can share emotions, understand others’ perspectives, and are motivated to provide help or comfort, they establish better social support (such as better-quality friendship; Denham et al., 1990; Zhou et al., 2002) and do less harm to other people (Lovett & Sheffield, 2007; Rieffe & Terwogt, 2006).

Whether the protective effect of empathy on psychopathology can also be extended to the population with hearing loss remains an unexplored topic. Although children with hearing loss have been found to differ from their TH peers in some empathic skills, no studies have examined how these differences in empathic skills may associate with their psychological wellbeing. Considering that the prevalence rates of internalizing and externalizing behaviors in deaf and hard-of-hearing children are 4 to 14 percentage points higher than the rates in TH children (e.g., Fellinger et al., 2008; Theunissen et al., 2014; Van Eldik et al., 2003), further investigations on the role of empathy in the development of children with hearing loss may carry important rehabilitative implications (see Schonert-Reichl et al. (2012) and Teding van Berkhout and Malouff (2016) for the effectiveness of empathy training programs).

## The Present Study

In this four-wave study, we focused on the preschool years because it is a crucial period for learning various social and emotional skills, and thus an important window for understanding early difficulties in social-emotional functioning experienced by children with a CI (Pahl & Barrett, 2007). With a longitudinal design, we could determine whether these children showed an early delay and remained low over time, or experienced elevated difficulties with increasing age due to limited input from the social environment.

Our first goal was to examine the levels and developmental trajectories of four empathic skills (affective empathy, attention to others’ emotions, prosocial actions, and acknowledgment of others’ emotions) in 1- to 5-year-old children with a CI and TH children by measuring these skills at four time points with a 12-month interval. Regarding the overall levels of empathic skills, we expected children with a CI to score similarly on affective empathy, higher on attention to emotions, and lower on prosocial actions and emotion acknowledgment than their TH peers (Dirks et al., 2017; Netten et al., 2015; Wang et al., 2019; Wang et al., 2016). Regarding the developmental trajectories of these skills, thus far there is no evidence that supports a different empathy development in children with hearing loss. Moreover, the maturity principle (Roberts & Mroczek, 2008; Roberts et al., 2008) suggests that psychologically adaptive functions would generally increase with age, whereas maladaptive functions would show age-related declines. Considering this principle and the empathy maturation model of Hoffman (1990), we expected an increase with age in attention to others’ emotions, prosocial actions, and emotion acknowledgment in preschool children, regardless of their hearing status. We also expected a decrease in the level of affective empathy with age as children become better at attending to other people’s emotions rather than their own arousal, in the two groups alike.

Our second goal was to examine the longitudinal effects of empathic skills on early symptoms of psychopathology (i.e., internalizing and externalizing behaviors) in children with a CI and TH children. Based on the longitudinal studies on children with typical development (Hastings et al., 2000; Zhou et al., 2002), we expected all empathic skills to have a negative association with internalizing/externalizing behaviors in TH children. We did not make specific hypotheses for children with a CI given the lack of studies on the association between empathic skills and psychopathology in children with hearing loss.

## Methods

### Participants and Procedure

A total of 343 children participated in this study (Table 1). Of these, 71 children had a CI, and the other 272 children were TH. They were between 1 and 5 years old at Time 1 (*M* = 3.16, *SD* = 1.14). The children with a CI were recruited through hospitals and family counseling services in the Netherlands and the Dutch-speaking areas of Belgium. The TH children were recruited through day-care centers and primary schools in the Netherlands. None of the children had additional disabilities or diagnoses other than hearing loss. The children with a CI were diagnosed with congenital or prelingual severe-to-profound bilateral hearing loss, and received at least one CI (37 children received bilateral implantation). All of the children with a CI entered a tailored rehabilitation program following implantation for aural-verbal training, technical support for the device, and specialized playgroups. See Table S1 for an explanation on sample size estimation.

Parents were asked to fill out questionnaires on social-emotional development at four time points. The average duration of the time intervals was 13.14 (*SD* = 3.08), 11.97 (*SD* = 1.22), and 11.97 (*SD* = 1.07) months between Time 1 and Time 2, Time 2 and Time 3, and Time 3 and Time 4, respectively. Other information, such as household income, parent’s educational level, age at implantation, and hearing history, was acquired from parents and/or medical records. Besides, children’s fine motor development at Time 1 was used as an indicator of their cognitive development, given the difficulty to obtain reliable IQ scores in children as young as one year and the close link between fine motor skills and cognitive skills, such as executive functioning (Roebers et al., 2014) and reasoning (Martzog et al., 2019; Pitchford et al., 2016). The fine motor scale (30 items) of the standardized Dutch-version *Child Development Inventory* (*CDI*) was used (Ireton & Glascoe, 1995). Parents rated on all 30 items whether their children showed a certain fine motor skill (0 = no; 1 = yes). As Table 1 shows, at Time 1 the children with a CI did not differ from the TH children in their age, *t* = 1.42, *p* = 0.155, gender distribution, χ^2^ = 2.35, *p* = 0.126, fine motor development, *t* = 1.19, *p* = 0.235, parental education level, *t* = -0.10, *p* = 0.924, or net household income, *t* = 1.28, *p* = 0.216.

The study protocol was approved by the Medical Ethics Committee of the Leiden University Medical Center (Approval number P08.140/SH/sh). Informed consent forms were signed by the parents of all children. This study is part of a large-scale longitudinal project on the social-emotional development of children with communicative difficulties, including children with a CI, children with Autism Spectrum Disorder, and children with Developmental Language Disorder (Broekhof et al., 2015; Ketelaar et al., 2010, 2012, 2013, 2015, 2017; Li et al., 2020; Netten et al., 2018; Rieffe & Wiefferink, 2017; Wiefferink et al., 2012, 2013). Part of the data on empathy (Time 1) and on psychopathology (Time 1 to 3) in children with a CI and TH children was previously reported by Ketelaar and coleagues (2013, 2017) and Netten and colleagues (2018), respectively.

## Materials

### Parent Reports

The *Empathy Questionnaire* was designed to measure young children’s empathic behaviors in daily life (Rieffe et al., 2010). It was rated by parents to indicate the extent to which each item reflected their child’s behaviors during the past two months (0 = never; 1 = sometimes; 2 = often), and it includes three subscales: affective empathy (6 items; e.g., “When another child cries, my child gets upset too”), attention to emotions (7 items; e.g., “When another child is angry, my child stops his own play to watch”), and prosocial actions (6 items; e.g., “When another child starts to cry, my child tries to comfort him/her”). Internal consistency was adequate across time for affective empathy (66 ≤ α ≤ 0.78), attention to emotions (72 ≤ α ≤ 0.82), and prosocial actions (0.66 ≤ α ≤ 0.76; see Table 2 for the internal consistency for all measures per time point). The internal consistencies were relatively lower for affective empathy at Time 3 and prosocial actions at Time 4 (α = 0.66) due to little variance in the scoring of some items (i.e., a near-floor effect for affective empathy and a near-ceiling effect for prosocial actions).

The emotion acknowledgment subscale of the *Emotion Expression Questionnaire* was used to measure children’s ability to acknowledge their parents’ emotions (Rieffe et al., 2010). Parents rated on a 5-point scale (1 = almost never; 5 = almost always) the extent to which their children could understand their emotions (6 items; e.g., “Does your child understand when you are angry?”). Internal consistency was good across time (0.70 ≤ α ≤ 0.78).

The *Early Childhood Inventory-4* (ECI-4) is a widely-used questionnaire rated by parents for assessing Diagnostic and Statistical Manual of Mental Disorders (4th ed.; DSM-IV) symptoms (Sprafkin et al., 2002). It can be scored according to the screening cut-off (dichotomous) or according to the symptom severity (on a 4-point scale: 0 = never; 3 = very often). We used the severity scores to reflect the severity of internalizing and externalizing symptoms. For measuring the level of internalizing behaviors, we used the subscales for major depressive disorder (6 items) and anxiety disorder (14 items, including generalized anxiety disorder, separation anxiety disorder, and social anxiety disorder). For measuring the level of externalizing behaviors, we used the subscales for peer conflict (10 items), oppositional defiant disorder (8 items), and conduct disorder (10 items). The severity scores of each subscale were summed to calculate final scores for internalizing/externalizing behaviors. Internal consistency was good across time for internalizing behaviors (0.78 ≤ α ≤ 0.88), and for externalizing behaviors (0.87 ≤ α ≤ 0.92). While the *ECI-4* was designed for children aged three to six years, its reliability in assessing children between one and three years old has been shown to be good (e.g., Ellis et al., 2004; Li et al., 2020; Maoz et al., 2014; Netten et al., 2018). For interpretation purposes, T scores of the symptoms are reported in Table S2.

## Analysis and Results

Statistical analyses were performed using SPSS version 25 (SPSS Inc., Chicago, IL, USA). Graphs were made in R version 3.6.3 (*Ggplot2* package). Considering the two-level structure in our data, i.e., time points (level 1) nested within participants (level 2), we used linear mixed models (LMMs) with maximum likelihood estimation to analyze the longitudinal data. LMMs allow the dependency within the data to be accounted for. A predictor variable was regarded as having a significant contribution to the model when its 95% confidence interval (95% CI) did not include the value zero.

## Missing Values and Multiple Imputation

At Time 1, missing scores were found on *Empathy Questionnaire* (0 CI, 4 TH), *Emotion Expression Questionnaire* (0 CI, 2 TH), *ECI-4* (4 CI, 16 TH), fine motor development (16 CI, 27 TH), parental education level (18 CI, 38 TH), and net household income (31 CI, 95 TH). The Little’s MCAR test showed that data at Time 1 were missing completely at random, χ^2^ = 20,955, *df* = 21,054, *p* = 0.684. To handle the missing data at Time 1, we used multiple imputations (MI). The MI technique fills in missing data according to participant characteristics and relations observed in the data with other participants (Azur et al., 2011; Schafer & Graham, 2002; van Ginkel et al., 2019), thus increasing statistical power and reducing biases caused by missing data (Donders et al., 2006; Netten et al., 2017). The following variables were included for the estimation of missing values: age, gender, hearing status, fine motor development, parental education level, net household income, and outcomes on the three parent reports. Ten imputations were performed (Sterne et al., 2009), and pooled results are reported.

Only missing data at Time 1 were imputed because LMMs can handle missing follow-up data points of a participant as long as values are missing (completely) at random (Twisk et al., 2013). Therefore, participants who had missing data at Time 2, 3, or 4 were still included in the analyses. Missing data were found at Time 2 (25 CI and 164 TH children), Time 3 (25 CI and 176 TH children), and Time 4 (44 CI and 204 TH children). For 38 children with a CI (54%) and 92 TH children (34%), data were available for at least three time points. The Little’s MCAR test showed that the values across the four time points were not missing completely at random (*p* < 0.001). Yet, the missingness of the values was dependent on observed characteristics of the participants, i.e., time points of participation and diagnosis. Participants without Time 2 data also did not have data from Time 3 and Time 4; and the drop-out was more frequently observed in the TH group (see Table S3 for an overview of the amount of missing data). This was because children with a CI visited hospitals or counseling services regularly and could be followed up there. Given that the missingness could largely be explained by observed data, we assumed that values were missing at random and proceeded with LMMs. Children with and without missing data points did not differ in age at Time 1, *t* = -1.26, *p* = 0.208, gender distribution, χ^2^ = 1.29, *p* = 0.256, fine motor development, *t* = -0.99, *p* = 0.324, and parental education level, *t* = -1.16, *p* = 0.247. Yet children who participated in all waves had higher net household income than those with missing data points, *t* = -2.59, *p* = 0.010.

## Descriptive Statistics

Table 2 shows the total scores and standard deviations for the variables per group at each time point and the independent *t*-statistics for group comparison. Based on parent reports, the children with a CI exhibited fewer prosocial actions than their TH peers at Time 3, *t* = 1.99, *p* = 0.047, and Time 4, *t* = 2.02, *p* = 0.044. No other group differences were found. See Tables S4 and S5 for correlations between study variables and between study variables and hearing-related factors. Fig. S1 shows graphic representations of individual variations at the four time points.

## Levels and Developmental Trajectories of Empathic Skills

Via a formal model-fitting procedure of LMM, increasingly more complex models were fitted to the data. By using the total score of each empathic skill, respectively, as the dependent variable, we started by fitting an unconditional means model which included only a fixed and random intercept as a baseline. Then, we included age (centered) to the model and examined two trends of change: linear and quadratic. A random-slope effect for age and a fixed effect for gender (0 = boys; 1 = girls) were added to the best age model, which did not improve the model fits and thus are not reported here. Finally, group membership (0 = TH; 1 = CI) and its interaction with age were added, to examine if there were group differences in the overall level of each empathic skill and its developmental course. The -2 log likelihood (-2*LL*) values were used to compare between the model fits (the stacking procedure suggested by Wood and colleagues (2008) was used to obtained the -2*LL* values after multiple imputations). The likelihood ratio test was conducted to test whether the deviance in the -2*LL* values was significant. Preferred models should have significantly lower -2*LL* values. Best-fitting models are reported in Table 3.

**Fig. 1:**
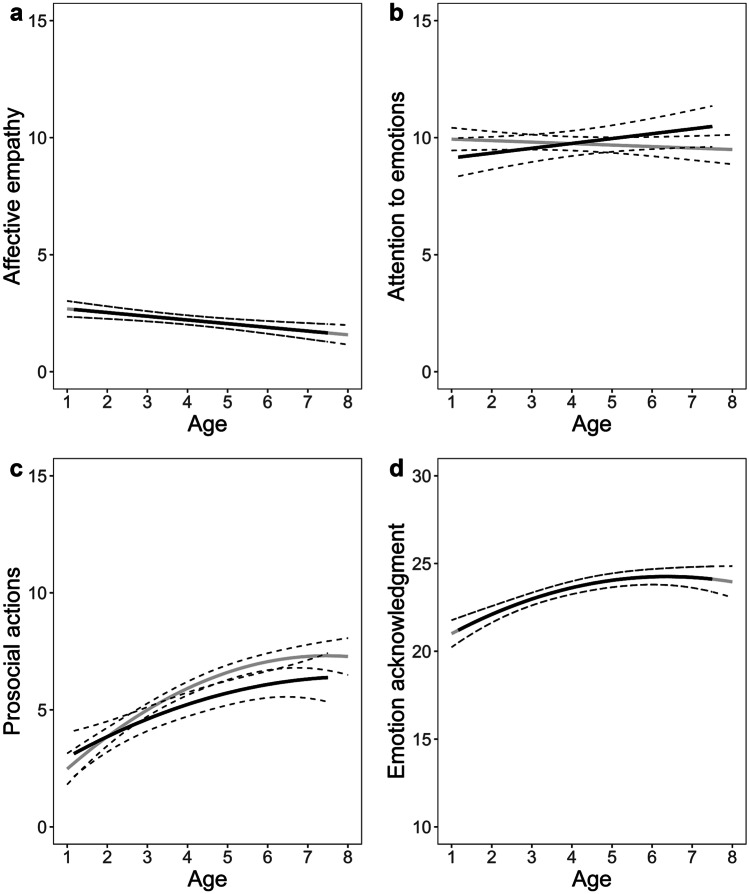
Longitudinal graphic representation of the predicted values based on the optimal fitting model for **a** affective empathy; **b** attention to others’ emotions; **c** prosocial actions; **d** emotion acknowledgment. *Note*. Lines for children with a cochlear implant are displayed in black, and lines for typically-hearing children are displayed in grey. Dotted lines represent 95% confidence interval

Affective empathy and attention to others’ emotions were both best explained by a linear age-model (Fig. 1a, b). Affective empathy decreased with age, *b* = -0.01, 95% *CI* [-0.02, -0.01], and no group differences appeared. Attention to others’ emotions was unrelated to age in TH children, *b* = 0.01, 95% *CI* [-0.02, 0.01], but increased with age in children with a CI as indicated by an interaction of group with age, *b* = -0.03, 95% *CI* [0.01, 0.05]. No other group effects were observed.

Prosocial actions and emotion acknowledgment were both best explained by a quadratic age-model. This indicates that prosocial actions and emotion acknowledgment increased with age and stabilized around the time when children entered primary schools (Fig. 1c, d). Yet, parents reported that children with a CI showed fewer prosocial actions than their TH peers across time, *b* = -0.55, 95% *CI* [-1.06, -0.03]. For emotion acknowledgment, there were no group differences.

## Longitudinal Effect of Empathy on Internalizing/Externalizing Behaviors

To investigate both between- and within-person effects of empathic skills on the development of internalizing/externalizing behaviors across time, we first calculated a mean score (between persons) and a change score (within persons), for each empathic skill. The mean score is represented by the overall mean score of the four measurement points per participant (i.e., a participant’s average level across time points). It was added to the model to examine how the development of psychopathological symptoms could be explained by the differences between participants in the level of a given empathic skill. The change scores indicate the deviations around this mean score (i.e., Time 1 – mean; Time 2 – mean; Time 3 – mean; Time 4 – mean), and were used to examine whether the development of psychopathological symptoms could be explained by within-person changes in the level of an empathic skill over time (Singer & Willett, 2003).

By using the frequency of internalizing and externalizing behaviors, respectively, as the dependent variable, we started with fitting a model with three control variables: age, gender, and group membership. In the next model, all the empathic skills (mean and change scores) were fitted to the model to check their unique contributions to internalizing/externalizing behaviors. Subsequently, we added the interaction terms between group and one of the empathic skills (mean and change scores), one skill at a time, to examine whether the effect of the skill differed between groups. The interaction terms would be included in the final model if adding them significantly improved the model fit. Table 4 shows the best-fitting models for internalizing and externalizing behaviors.

In the model for internalizing behaviors, we observed effects of affective empathy (mean score), *b* = 0.63, 95% *CI* [0.44, 0.82], affective empathy (change score), *b* = 0.23, 95% *CI* [0.05, 0.41], and attention to others’ emotions (change score), *b* = 0.19, 95% *CI* [0.03, 0.35]. This indicates that children with a higher mean level of affective empathy, and children with a larger increase in their affective empathy and attention to emotions over time, showed an increase in internalizing behaviors. The addition of group interaction terms did not further improve the model fits. This suggests that the effects of the empathic skills in the two groups had similar strengths across time.

In the model for externalizing behaviors, we observed effects of affective empathy (mean score), *b* = 0.35, 95% *CI* [0.02, 0.67], and emotion acknowledgment (mean score), *b* = -0.21, 95% *CI* [-0.40, -0.01]. This suggests that children with higher mean level of affective empathy and a lower mean level of emotion acknowledgment showed an increase in externalizing behaviors. Adding group interaction terms did not improve the model fits, suggesting similar strength for empathic effects in the two groups across time.

Given the unequal group sizes of children with a CI and TH children, we repeated the analyses on a sub-sample of 71 children with a CI and 71 TH children randomly selected from the full sample. The directions of results remained unchanged, although the significant contributions of some predictor variables were not observed in the smaller sample (see Tables S6 and S7).

## Discussion

Current knowledge about children’s development regarding empathy is largely based on studies of children with typical development. This four-wave study is among the first to longitudinally investigate the development of empathy and its effects on early symptoms of psychopathology in children with a CI and children with typical hearing. Notably, differences between the groups were not often observed. This suggests that the empathy development of children with a CI was broadly on par with their TH peers. When parents reported on their child’s level of affective empathy, no group differences appeared. In both groups, affective empathy decreased with age, and higher levels of affective empathy were related to more psychopathological (i.e., internalizing and externalizing) symptoms. The overall level of attention to others’ emotions were not different between the groups, whereas the trajectories of the two groups differed: A stable trend in TH children but an increasing trend in children with a CI were observed over time. In the two groups alike, children who became increasingly attentive to others’ emotions over time were more likely to develop internalizing behaviors. Prosocial actions were more often reported in TH children than in children with a CI. Over time, an increase in prosocial actions was observed in both groups, which stabilized after children entered primary schools. Yet this trend was unrelated to the development of psychopathology. The level of emotion acknowledgment did not differ between the groups. Like prosocial actions, emotion acknowledgment increased with age and became stable at the beginning of school age in both groups. Higher levels of this skill contributed to a decrease in externalizing symptoms. Below, we will discuss the implications of these findings in greater detail.

## Affective Empathy

Children with a CI and their TH peers were similar in the levels and developmental trajectories of affective empathy. In line with the theory proposed by Hoffman (1990), affective empathy declined with age in both groups of this study. Considering that affective empathy – also called emotion contagion (Hatfield et al., 1993) – is a basic building block of empathy, this result is not surprising. Affective empathy involves a primitive arousal mode, which is thought to be present at birth and prewired in the mirror neuron system in the brain (Decety & Jackson, 2004; Eisenberg et al., 2006; Engen & Singer, 2013). When the level of such arousal is too high, children tend to focus on the emotional reaction triggered in themselves and to alleviate their own arousal, rather than turning their attention to the person actually experiencing the emotion (Eisenberg et al., 2006; Hoffman, 1990). With an improved self-other differentiation, children experience a more moderate level of personal arousal. The decreasing trend we found in the current study appears to follow this developmental trajectory, driven by the need to keep a moderate level of personal arousal – thus able to react adaptively while sharing others’ emotions.

For this reason, children in this study who instead retained higher levels of affective empathy, or showed an increase in the levels of affective empathy over time, were at greater risk of developing psychopathological symptoms, including both internalizing and externalizing symptoms. These children experienced a higher level of personal arousal when witnessing others’ emotions, which could lead to self-oriented responses to the emotions and prevent them from responding adaptively to the situation (Eisenberg et al., 2006; Rieffe et al., 2010). An inward processing of emotions, also when triggered by others’ affective states, and incompetent reactions to the external world, are characteristic of internalizing and externalizing behaviors.

## Attention to Others’ Emotions

In the current study, the level of attention to others’ emotion remained stable in TH children, but increased in children with a CI over time. According to Hoffman (1990), children start to direct more attention to others’ emotions from the age of one year. At this age, children know better that what others are feeling is different from their own affective state, thus they can observe others’ emotional displays without experiencing too much personal arousal. In our study, children at the first measurement had a mean age of three years. The stable trend we found in TH children suggests that from the age around three years TH children become more skilled with grasping emotional information and the processing is more automatic to them. Thus, attention beyond sufficient level is unnecessary for TH children.

This result showed that, like affective empathy, attention to emotions may not be the more the better. While directing attention to others helps a person understand others’ emotions, paying too much attention to others’ emotional displays may leave the person with little mental energy to channel to other things in the surroundings or to evaluating a proper response. Following the same line of reasoning, the increasing level of attention to others’ emotions we observed in children with a CI may reflect elevated vigilance or sensitivity to emotions (Pérez-Edgar et al., 2010). Alternatively, children may recruit increasingly more attentional resources because they find emotional events become more challenging to process (Wild et al., 2012). Whichever is the case, increased attention over time may reflect that children experience more effortful processing of others’ emotions with age.

This group difference in the developmental trend of attention could be alarming, because our results also showed that children who became increasingly attentive to others’ emotions over time were more likely to develop internalizing symptoms. The more effortful processing of others’ emotions could lead to more difficult coping with negative emotions observed in other people for these children. Although in this study we did not find children with a CI to develop more internalizing behaviors than their TH peers during the preschool years, the increasing levels of attention to emotions observed in children with a CI highlight the need to study these children’s empathic maturation and psychopathological symptoms at later stages of life. Moreover, it should be noted that only the change scores of attention to emotions, but not the mean scores, contributed to the development of internalizing behaviors in our analysis. This indicates that changes in attention level is a signal that children are facing difficulties processing others’ emotions and may need support.

## Prosocial Actions and Emotion Acknowledgment

Prosocial actions and emotion acknowledgment both increased with age and stabilized when the two groups of children entered primary schools. Unlike affective empathy and attention to others’ emotions, which may involve only “sit and watch,” prosocial actions and emotion acknowledgment require proactive responses and understanding of emotions and social rules. Our results suggest that children keep developing these skills throughout preschool years until around the beginning of school age, when they start to recognize others’ basic emotions and show the intention to comfort or help other people in a more stable manner.

However, despite the similar developmental trajectories, children with a CI were rated lower on prosocial actions than their TH peers. Such a group difference may be best explained by children with a CI’s limited incidental learning (Netten et al., 2015) and Theory of Mind (ToM) ability (Ketelaar et al., 2012). To react prosocially to others’ emotions, children have to know why the other person is experiencing an emotion, and how to benefit the person in a socially appropriate way. This requires ToM, i.e., the ability to understand, explain, and predict other people’s mental states, which guides children’s (emotional) behaviors (Goldman, 2012; Wellman & Liu, 2004). Yet, such an ability could only be obtained within a social context where children learn the why and how through observing, overhearing, and participating in social interactions (Rieffe et al., 2015; Saarni, 1999). As described earlier, children with a CI experience a lower quantity and quality of social interactions in the predominantly hearing social environment. Many opportunities for learning prosocial actions are thus missed during early childhood.

While the development of prosocial actions was unrelated to psychopathological symptoms, higher levels of emotion acknowledgment were associated with fewer externalizing behaviors, in the two groups alike. When children improve the ability to acknowledge others’ emotions, they may better theorize other people’s states of mind and more appropriately interpret the situation they are in (Brüne, 2005; Cassidy et al., 2003; Lane et al., 2010). A more thorough evaluation of social situations may thus help children react to the external world in a more adaptive manner. However, it should be noted that children with a CI are known for their ToM problems (Ketelaar et al., 2012; Peterson, 2016; Peterson & Siegal, 2000). When these children are required to theorize more complex mental states in others beyond the basic emotions examined in this study, emotion acknowledgment might start to be challenging. This again underscores the importance of giving children with a CI an accessible social environment because the social context is required for learning emotional knowledge.

## Limitations and Future Research

The current study has the strength of examining different empathic skills in children with a CI and TH children using a four-wave longitudinal design. It is among the first to investigate empathy development in children with hearing loss, and to show that maladaptive empathic responses could be a risk factor for children with typical and atypical development alike, when they are studied over time. Our outcomes stress the idea that each empathic skill may be related differently to behavior, and therefore needs to be examined separately.

However, some considerations are needed when interpreting the results. First, further investigations will be necessary to understand how much the current outcomes can be generalized to other groups of children with hearing loss, such as those with mild-to-moderate or unilateral hearing loss. Deaf and hard-of-hearing children represent a highly heterogeneous group, and children with a CI are usually the ones that receive more intensive rehabilitative training, and have better auditory and oral language performance. Also, we had fewer children with a CI than TH children in our sample. When we matched the sample size between children with a CI and TH children, thus having a smaller total sample size, the effect of empathy on externalizing behaviors was no longer observed, while the effect on internalizing behaviors remained robust (see Table S7). This implies that the composition of the study sample may potentially affect our findings about externalizing behaviors. Therefore, to examine the generalizability of the current results, future studies are suggested to recruit a larger clinical group, and to include deaf or hard-of-hearing children with different hearing, family, or language backgrounds given that the variability in hearing conditions theoretically may affect the development of empathic responses.

Second, it should be noted that we used only parent reports. Past studies have shown that parent–child agreement on children’s emotional competence and psychopathology is lower when the child has clinical conditions than when the child is typically developing (Barbosa et al., 2002; Johnson et al., 2009), and the self-reported level of internalizing behaviors is often higher than parent-reported level (Anmyr et al., 2012; Hope et al., 1999). Therefore, collecting data from different methods, such as real-life playground observations or in vivo experiments, is suggested for future research to increase ecological validity.

Third, the questionnaires used in the current study were designed for young children. This means that only empathic skills that involve basic emotions and simple social interactions were considered. Therefore, the stabilizing developmental trends and small group differences found in this study should be interpreted with caution. Further studies are needed to understand how children with a CI develop to show empathy to more complex emotions (e.g., embarrassment and shame) and social situations (e.g., what to do when others are having arguments). Moreover, it should be noted that the *ECI* was designed for children between three to six years old. Although previous studies have shown its good reliability in assessing children below three years old, and the majority (80%) of the children in this study were three years old or older from Time 2, this limitation should be taken into account when interpreting the results of psychopathological symptoms at Time 1.

## Conclusions

The present four-wave study paints a largely positive picture of young children with a CI. These preschool children with a CI and their TH peers in general had similar levels of empathic skills and developed these skills with similar trajectories. However, parents reported that children with a CI were increasingly more attentive to others’ emotions over time and carried out fewer prosocial actions across time, compared to TH children. Children with a CI may need more opportunities for social access to learn to process others’ emotions less effortfully and react to other people more prosocially.

Moreover, the effects of empathic skills on early symptoms of psychopathology were similar in the two groups of children. This indicates that intervention programs for psychopathology that tackle children’s empathic responses could be beneficial for children with a CI and TH children, alike. On one hand, children who show a strong affective response and become increasingly attentive to other people’s emotional displays may need extra support to develop more adaptive behaviors. Such an intervention may be particularly relevant to children with a CI, given their increasing level of attention to emotions during preschool years. On the other hand, training children to acknowledge other people’s emotions may help them understand emotional situations better, thus decreasing externalizing symptoms.

Taken together, this study demonstrated the necessity that children with a CI are provided with more opportunities to acquire emotional knowledge in daily social life. This may be achieved by making social interactions more accessible to these children through, for example, multiple communication means (e.g., oral language supported by sign language) and a more inclusive environment where these children’s needs are addressed. Including the emotional domain in rehabilitation programs for children with a CI is also suggested.

**Table 1:** Participant characteristics

	CI	TH
Number of children at Time 1	71	272
Gender, female, n (%)	28 (39%)	135 (50%)
Age at Time 1, years, mean (SD)	3.21 (1.22)	3.25 (1.13)
Fine motor development^a^, mean (SD)	19.95 (6.92)	20.83 (6.51)
Parental education^b^, mean (SD)	3.51 (.69)	3.46 (0.77)
Net household income^c^, mean (SD)	3.63 (1.15)	3.91 (0.99)
Age at implantation, years, mean (SD)	1.37 (.73)	
Duration of using CI at Time 1, years, mean (SD)	1.54 (1.07)	
Type of amplification		
Unilateral cochlear implantation	14 (20%)	
Bimodal fitting	16 (23%)	
Bilateral cochlear implantation	37 (52%)	
Unknown	4 (5%)	
Preferred mode of communication		
Spoken language only, n (%)	19 (27%)	
Sign-supported Dutch, n (%)	34 (48%)	
Dutch sign language, n (%)	7 (10%)	
Combination of communication modes, n (%)	8 (11%)	
Unknown	3 (4%)	

*CI* cochlear implant, *TH *typically hearing

^a^Scores ranged between 0 and 30

^b^Parental education level: 1, no/primary education; 2, lower general secondary education; 3, higher general secondary education; 4, college/university

^c^Net household income: 1, less than €15,000; 2, €15,000 – €30,000; 3, €30,000 – €45,000; 4, €45,000 – €60,000; 5, more than €60,000

**Table 2:** Psychometric properties and total scores of study variables at each time point

	No.Items	Scale	Cronbach’s alpha	Mean (SE)^a^		
				CI	TH	*t* value^a^
Time 1						
Affective empathy	6	0-2	0.69	2.59 (0.28)	2.24 (0.12)	-1.30
Attention to emotions	7	0-2	0.72	9.32 (0.33)	9.70 (0.16)	1.08
Prosocial actions	6	0-2	0.76	4.45 (0.31)	5.05 (0.16)	1.73
Emotion acknowledgment	6	1-5	0.76	22.65 (0.41)	22.88 (0.21)	0.50
Internalizing behaviors	20	0-3	0.78	2.78 (0.39)	2.87 (0.19)	0.21
Externalizing behaviors	28	0-3	0.87	8.13 (0.75)	7.74 (0.32)	-0.52
Time 2						
Affective empathy	6	0-2	0.78	2.36 (0.32)	2.43 (0.23)	0.18
Attention to emotions	7	0-2	0.75	10.05 (.37)	9.91 (0.26)	-0.30
Prosocial actions	6	0-2	0.71	5.55 (0.37)	5.92 (0.23)	0.86
Emotion acknowledgment	6	1-5	0.70	23.71 (0.50)	24.18 (0.30)	0.83
Internalizing behaviors	20	0-3	0.80	3.22 (0.49)	3.39 (0.39)	0.27
Externalizing behaviors	28	0-3	0.89	9.48 (0.96)	7.58 (0.60)	-1.73
Time 3						
Affective empathy	6	0-2	0.66	2.3 (0.30)	2.04 (0.20)	-0.74
Attention to emotions	7	0-2	0.74	9.91 (0.39)	9.59 (0.27)	-0.66
Prosocial actions	6	0-2	0.71	5.88 (0.33)	6.69 (0.23)	1.99*
Emotion acknowledgment	6	1-5	0.78	24.04 (0.52)	24.32 (0.34)	0.47
Internalizing behaviors	20	0-3	0.81	3.39 (0.63)	3.33 (0.37)	-0.10
Externalizing behaviors	28	0-3	0.87	8.66 (0.86)	7.59 (0.56)	-1.08
Time 4						
Affective empathy	6	0-2	0.74	2.02 (0.43)	1.82 (0.21)	-0.46
Attention to emotions	7	0-2	0.82	10.65 (0.48)	9.84 (0.35)	-1.28
Prosocial actions	6	0-2	0.66	5.93 (0.41)	6.90 (0.26)	2.02*
Emotion acknowledgment	6	1-5	0.72	24.45 (0.54)	24.28 (0.36)	-0.26
Internalizing behaviors	20	0-3	0.88	4.93 (1.18)	4.49 (0.62)	-0.03
Externalizing behaviors	28	0-3	0.92	10.50 (1.58)	7.86 (0.78)	-1.68

*CI* children with a cochlear implant, *TH *typically-hearing children

* p < 0.05 between children with a CI and TH children

^a^Multiple imputations were applied at Time 1

**Table 3:** Regression weights [95% CI] for explaining the developmental trajectories of empathic skills

Parameter	Affective empathy	Attention to others’ emotions	Prosocial actions	Emotion acknowledgment
Age linear	**-0.01 [-0.02, -0.01]**	-0.01 [-0.02, 0.01]	**0.06 [0.05, 0.07]**	**0.04 [0.02, 0.05]**
Age quadratic	-	-	**-0.001 [-0.001, -0.0003]**	**-0.001 [-0.001, -0.0003]**
Group	-	0.15 [-0.48, 0.77]	**-0.55 [-1.06, -0.03]**	-
Group x Age	-	**0.03 [0.01, 0.05]**	-0.01 [-0.03, 0.004]	-

Group was coded as 0, typically hearing; 1, cochlear implant. Significant effects are bolded

**Table 4:** Regression weights [95% CI] of empathic skills (mean and change scores) for predicting internalizing/externalizing behaviors

Parameter		Internalizing	Externalizing
Age		**0.06 [0.04, 0.07]**	**0.04 [0.02, 0.06]**
Gender		0.42 [-0.21, 1.06]	-1.07 [-2.17, 0.02]
Group		-0.26 [-1.01, 0.48]	0.44 [-0.83, 1.71]
Affective empathy	Mean	**0.63 [0.44, 0.82]**	**0.35 [0.02, 0.67]**
	Change	**0.23 [0.05, 0.41]**	0.19 [-0.10, 0.48]
Attention to emotions	Mean	0.07 [-0.08, 0.22]	0.15 [-0.10, 0.41]
	Change	**0.19 [0.03, 0.35]**	-0.09 [-0.34, 0.16]
Prosocial actions	Mean	-0.16 [-0.32, 0.004]	-0.01 [-0.29, 0.27]
	Change	-0.06 [-0.22, 0.09]	0.06 [-0.19, 0.30]
Emotion acknowledgment	Mean	-0.11 [-0.22, 0.0003]	**-0.21 [-0.40, -0.01]**
	Change	-0.05 [-0.17, 0.07]	-0.12 [-0.31, 0.08]

Gender was coded as 0, boys; 1 girls. Group was coded as 0, typically hearing; 1 cochlear implant. Significant effects are bolded

## Supplementary Information

Below is the link to the electronic supplementary material.

Supplementary file1 (PDF 597 KB)

## Data Availability

Following the policy of the Unit of Developmental and Educational Psychology, Leiden University, the dataset and associated information used in the current study will be shared publicly on the archiving platform DataverseNL (https://dataverse.nl/) once the manuscript is accepted.
