# Supplementary material for: The Developmental Trajectory of Empathy and Its Association with Early Symptoms of Psychopathology in Children with and without Hearing Loss
Source: Res Child Adolesc Psychopathol. 2021 Apr 7;49(9):1151–64. doi: 10.1007/s10802-021-00816-x (PMC8322017; doi:10.1007/s10802-021-00816-x)
Supplement: Supplementary file 1 — Supplementary file1 (PDF 597 KB) [file 10802_2021_816_MOESM1_ESM.pdf]

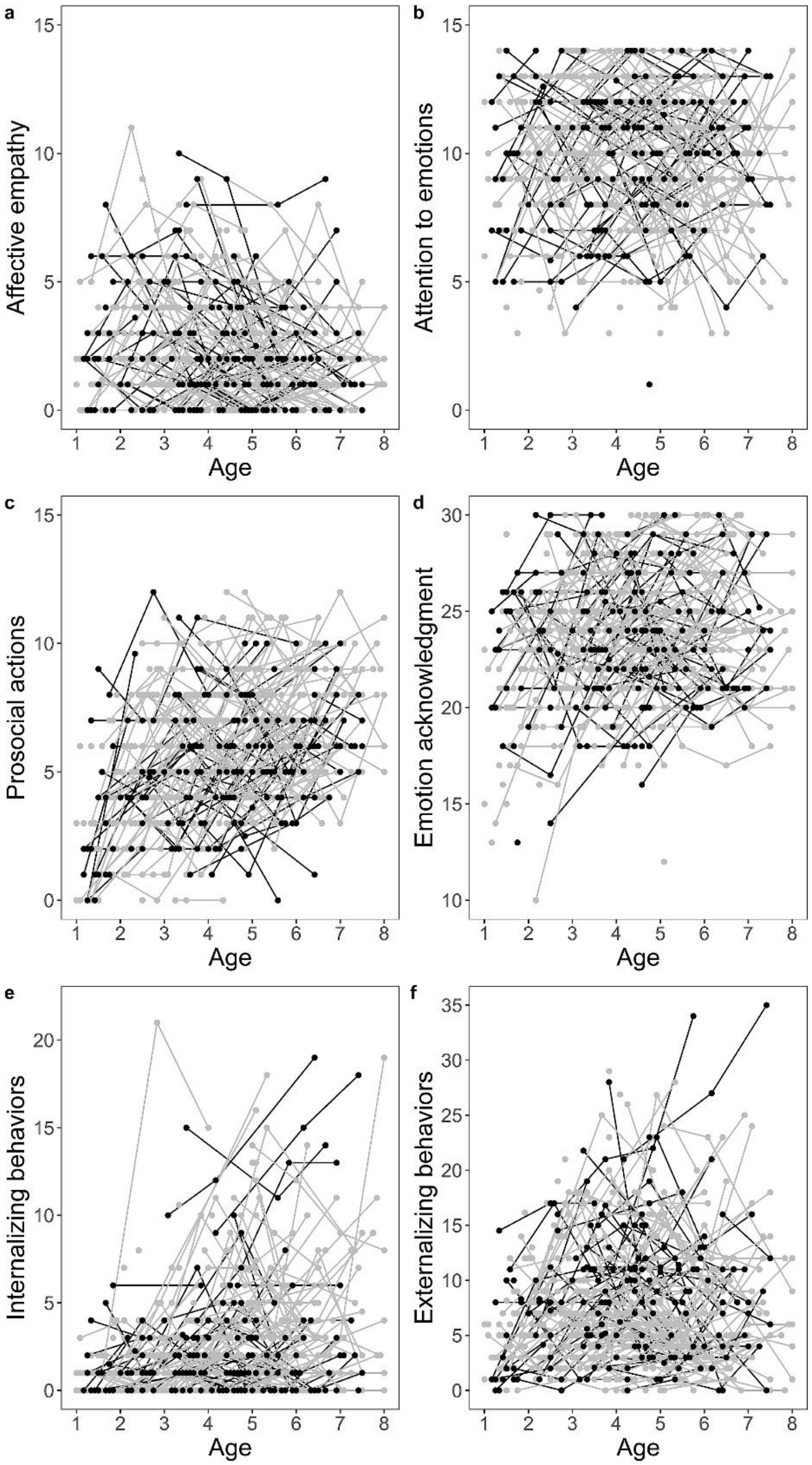

Fig S1. Longitudinal graphic representation of age at the four time points of **1a.** affective empathy; **1b.** attention to others' emotions; **1c.** prosocial actions; **1d.** emotion acknowledgment; **1e.** internalizing behaviors; **1f.** externalizing behaviors. Each participant is presented by an individual line and each time point is presented by a point. Children with a cochlear implant are displayed in black, and typically-hearing children in grey.

Table S1. *Sample size justification.*

Analysis	Explanation
Power analysis for the larger project	An a priori power analysis was conducted for the larger project that embedded this study. It showed that to observe a medium-sized effect (effect size = .35, power = .80, alpha = .05), a minimum total sample size of 216 children would be needed for analyses with four repeated measures and two groups. Note that the analysis was done based on a repeated measure ANOVA design. Later we changed to mixed models for analyzing the data because mixed models can account for the dependency within the data and handle missing or unbalanced data.
Power analysis for the present study	We did not conduct a power analysis specifically for this study because the study was based on the data already collected. Yet, a simulation analysis was conducted via the Optimal Design program (Version 3.01; Raudenbush et al., 2011), to understand the sample size needed for detecting the effect of diagnosis group in multilevel models. It showed that in the case where each participant has two waves of data, an effect of group can be detected with a power ≥ .80 when the total number of participants is ≥ 150 (alpha = .05; effect size = .35).
Estimation of the control group size	Because (1) the larger project intended to include three clinical groups (hearing loss, autism, developmental language disorder) and a control group that can be matched with the three groups (e.g., the autistic sample was expected to be older than children with a CI, so we needed older control group as well), and (2) typically-developing children tend to have a higher drop-out rate, we included a larger control group size. We intended to avoid possible bias from selection and from estimation, so in this study we included all available data and chose to use mixed models.

Table S2. *Mean T scores (standard deviations) of internalizing and externalizing symptoms converted according to the Symptom Severity Profile of the Early Childhood Inventory (ECI-4).*

	CI	TH
**Time 1**		
Oppositional defiant disorder	51.00 (2.23)	51.33 (3.13)
Conduct disorder	55.66 (5.79)	54.61 (4.56)
Peer conflicts	55.21 (5.57)	54.50 (5.67)
Major depressive disorder	50.97 (1.73)	51.07 (1.22)
Separation anxiety disorder	52.97 (3.80)	52.77 (3.14)
Generalized anxiety disorder	52.09 (5.14)	51.29 (3.69)
**Time 2**		
Oppositional defiant disorder	52.10 (3.97)	51.68 (3.63)
Conduct disorder	57.05 (7.92)	55.19 (5.06)
Peer conflicts	56.43 (7.71)	54.34 (6.25)
Major depressive disorder	51.06 (1.44)	51.09 (1.12)
Separation anxiety disorder	53.62 (5.43)	52.85 (3.49)
Generalized anxiety disorder	52.85 (4.51)	52.89 (5.36)
**Time 3**		
Oppositional defiant disorder	51.59 (2.91)	51.69 (2.71)
Conduct disorder	56.14 (5.61)	54.55 (4.43)
Peer conflicts	54.55 (5.65)	53.81 (5.95)
Major depressive disorder	51.16 (1.96)	50.98 (1.01)
Separation anxiety disorder	53.77 (5.76)	53.01 (3.13)
Generalized anxiety disorder	-	-
**Time 4**		
Oppositional defiant disorder	54.07 (6.92)	52.35 (4.06)
Conduct disorder	56.30 (6.81)	54.79 (5.09)
Peer conflicts	55.63 (7.90)	53.84 (6.42)
Major depressive disorder	51.19 (1.00)	51.46 (2.79)
Separation anxiety disorder	55.33 (8.26)	54.19 (5.29)
Generalized anxiety disorder	-	-

Note. CI = children with a cochlear implant; TH = typically hearing children. T scores of 50 to 58 reflects low severity; 60-68 medium severity; ≥ 70 high severity. T scores for social anxiety disorder are not provided in the Symptom Severity Profile of the *ECI-4*. T scores for generalized anxiety disorder are not available for Time 3 and Time 4 because the conversion to T scores involved two items not directly

related to anxiety symptoms (but to symptoms of attention deficit hyperactivity disorder) which were thus not included in the data collection at Time 3 and Time 4.

Table S3. *An overview of amount of missing data at the four measurement points.*

	Participants		Missing			
	CI	TH	CI		TH	
			Count	%	Count	%
**Time 1**						
Age	71	272	0	0	0	0
Gender	71	272	0	0	0	0
Fine motor skills	55	245	16	22.5	27	9.9
Parental education	53	234	18	25.4	38	14.0
Household income	40	177	31	43.7	95	34.9
Affective empathy	71	268	0	0	4	1.5
Attention to emotions	71	268	0	0	4	1.5
Prosocial actions	71	268	0	0	4	1.5
Emotion acknowledgment	71	270	0	0	2	0.7
Internalizing	66	257	5	7	15	5.5
Externalizing	66	257	5	7	15	5.5
**Time 2**						
Age	46	108	25	35.2	164	60.3
Affective empathy	46	108	25	35.2	164	60.3
Attention to emotions	46	108	25	35.2	164	60.3
Prosocial actions	46	108	25	35.2	164	60.3
Emotion acknowledgment	46	108	25	35.2	164	60.3
Internalizing	47	99	24	33.8	173	63.6
Externalizing	47	99	24	33.8	173	63.6
**Time 3**						
Age	46	96	25	35.2	176	64.7
Affective empathy	46	96	25	35.2	176	64.7
Attention to emotions	46	96	25	35.2	176	64.7
Prosocial actions	46	96	25	35.2	176	64.7
Emotion acknowledgment	45	96	26	36.6	176	64.7
Internalizing	44	83	27	38.0	189	69.5
Externalizing	44	83	27	38.0	189	69.5
**Time 4**						
Age	27	67	44	62.0	205	75.4
Affective empathy	27	67	44	62.0	205	75.4

Attention to emotions	27	67	44	62.0	205	75.4
Prosocial actions	27	67	44	62.0	205	75.4
Emotion acknowledgment	27	68	44	62.0	204	75.0
Internalizing	27	63	44	62.0	209	76.8
Externalizing	27	63	44	62.0	209	76.8

Note. CI = children with a cochlear implant; TH = typically hearing children.

Table S4. *Pearson's correlations between study variables.*

	1.	2.	3.	4.	5.	6.
1. Age	-					
2. Affective empathy	-.106*	-				
3. Attention to emotions	.026	.350**	-			
4. Prosocial actions	.403**	.150**	.305**	-		
5. Emotion acknowledgment	.195**	.016	.262**	.365**	-	
6. Internalizing behaviors	.222**	.272**	.120*	.042	-.082	-
7. Externalizing behaviors	.095	.094	.067	.025	-.076*	.374**

* $p < .0083$; ** $p < .001$. Significance level was adjusted by the number of correlation analyses on each variable to $p < \alpha/6 = .0083$.

Table S5. *Correlations of the measures with hearing-related factors within children with a CI (partial correlation coefficients controlling for age were presented in parentheses)*.

	Age at implantation	Duration of using a CI	Type of amplification	Communication mode
Affective empathy	-.061 (-.012)	-.094 (.012)	.156 (.138)	.073 (.116)
Attention to emotions	-.020 (-.088)	.191* (.088)	.145 (.154)	.005 (.059)
Prosocial actions	.123 (.023)	.267** (-.023)	-.052 (-.027)	.073 (.080)
Emotion acknowledgment	-.010 (-.042)	.110 (.042)	.088 (.109)	-.056 (-.062)
Internalizing behaviors	-.018 (-.112)	.229* (.112)	.053 (.078)	.104 (.122)
Externalizing behaviors	-.037 (-.120)	.212* (.120)	.075 (.090)	-.004 (.016)

Note: Type of amplification was coded by the number of devices used (1 = only one CI; 2 = one CI and one hearing aid; 3 = two CIs). Communication mode was coded by the extent to which the child used spoken language as the major mode of communication (1 = sign language only; 2 = sign-supported Dutch; 3 = combination; 4 = spoken language only).

* $p < .0125$. ** $p < .001$. Significance level was adjusted by the number of correlation analyses on each measure to $p < \alpha/4 = .0125$.

Table S6. *Regression weights (standard errors) for explaining the developmental trajectories of empathic skills using the full sample (N = 343) and a sub-sample (N = 142, including all 71 children with CI, and 71 typically hearing children randomly selected from the full sample).*

Parameter	Affective empathy		Attention to others' emotions		Prosocial actions		Emotion acknowledgment	
	N = 343	*N* = 142	*N* = 343	*N* = 142	*N* = 343	*N* = 142	*N* = 343	*N* = 142
Age linear	**-.01 (.004)**	**-.02 (.005)**	-.01 (.006)	-.01 (.009)	**.06 (.005)**	**.04 (.006)**	**.04 (.006)**	**.03 (.008)**
Age quadratic	-	-	-	-	**-.001 (.0002)**	**-.001 (.0002)**	**-.001 (.0002)**	-
Group	-	-	.15 (.32)	.19 (.40)	**-.55 (.26)**	-	-	-
Group x Age	-	-	**.03 (.010)**	**.03 (.012)**	-.01 (.009)	-	-	-

Note. Group was coded as 0 = typically hearing, 1 = cochlear implant. Significant effects are bolded.

Table S7. *Regression weights (standard errors) of empathic skills (mean and change scores) for predicting internalizing/externalizing behaviors using the full sample (N = 343) and a sub-sample (N = 142, including all 71 children with CI, and 71 typically hearing children randomly selected from the full sample)*

Parameter		Internalizing		Externalizing	
		N = 343	*N* = 142	*N* = 343	*N* = 142
Age		**.06 (.007)**	**.06 (.009)**	**.04 (.01)**	**.04 (.02)**
Gender		.42 (.32)	.77 (.50)	-1.07 (.56)	-1.06 (.88)
Group		-.26 (.38)	-.60 (.50)	.44 (.66)	.65 (.87)
Affective empathy	Mean	**.63 (.10)**	**.73 (.16)**	**.35 (.16)**	-.06 (.28)
	Change	**.23 (.10)**	.19 (.11)	.19 (.15)	.15 (.19)
Attention to emotions	Mean	.07 (.08)	-.002 (.12)	.15 (.13)	.07 (.22)
	Change	**.19 (.08)**	**.24 (.09)**	-.09 (.13)	-.19 (.17)
Prosocial actions	Mean	-.16 (.08)	-.21 (.14)	-.01 (.14)	-.09 (.24)
	Change	-.06 (.08)	-.06 (.09)	.06 (.13)	.32 (.16)
Emotion acknowledgment	Mean	-.11 (.06)	-.18 (.10)	**-.21 (.10)**	-.22 (.18)
	Change	-.05 (.06)	-.03 (.07)	-.12 (.10)	-.13 (.12)

Note. Gender was coded as 0 = boys, 1 = girls. Group was coded as 0 = typically hearing, 1 = cochlear implant. Significant effects are bolded.
